# Correction: Superhydrophobic states of 2D nanomaterials controlled by atomic defects can modulate cell adhesion

**DOI:** 10.1039/d5cc90255j

**Published:** 2025-08-15

**Authors:** Manish K. Jaiswal, Kanwar Abhay Singh, Giriraj Lokhande, Akhilesh K. Gaharwar

**Affiliations:** a Department of Biomedical Engineering, Texas A&M University College Station TX-77843 USA gaharwar@tamu.edu; b Department of Biomedical Engineering, Texas A&M University College Station TX-77843 USA; c Center for Remote Health Technologies & Systems, Texas A&M University College Station TX-77843 USA

## Abstract

Correction for ‘Superhydrophobic states of 2D nanomaterials controlled by atomic defects can modulate cell adhesion’ by Manish K. Jaiswal *et al.*, *Chem. Commun.*, 2019, **55**, 8772–8775, https://doi.org/10.1039/C9CC00547A.

In **Fig. 1c**, the images labeled MoS_2_ (1 : 1) and MoS_2_ (1 : 4) were inadvertently reused from our previous publication,^1^ without proper attribution. The corrected caption is shown below. This does not affect the study's results or conclusions. We apologize for the oversight.

**Fig. 1** (c) SEM image shows a “flower”-like morphology of nanoassemblies of typical size 1.5–3 μm. No significant change in size or shape was observed due to changes in atomic vacancies for MoS_2_ (1 : 1, 1 : 2, 1 : 4 and 1 : 6 samples). Reproduced in part from ref. 1, *Adv. Mater*, Copyright 2017, with permission from Wiley.

The Royal Society of Chemistry apologises for these errors and any consequent inconvenience to authors and readers.

## Notes and references

1 M. K. Jaiswal *et al.*, *Adv. Mater.*, 2017, **29**, 1702037, DOI: 10.1002/adma.201702037.

